# Viral encephalitis related to adalimumab in a 16-year-old Crohn’s disease patient: case report and literature review

**DOI:** 10.3389/fimmu.2026.1759050

**Published:** 2026-03-10

**Authors:** Zao Liang, Qiuping Yang, Guixian Pan, Ximei Yang, Bingqing Liu, Wenting Gan, Ruoshi Yang, Hu Hao, Sitao Li, Yao Cai

**Affiliations:** 1 Department of Pediatrics, The Sixth Affiliated Hospital, Sun Yat-sen University, Guangzhou, Guangdong, China; 2 Guangdong Provincial Clinical Research Center for Digestive Diseases, Guangzhou, Guangdong, China; 3 Biomedical Innovation Center, The Sixth Affiliated Hospital, Sun Yat-sen University, Guangzhou, Guangdong, China

**Keywords:** Crohn’s disease (CD), infection, neurological complication, pediatric, tumor necrosis factor-alpha (TNF-α)

## Abstract

Tumor necrosis factor-alpha (TNF-α) inhibitors are extensively utilized in inflammatory bowel disease (IBD). Adalimumab (ADA), a TNF-α inhibitor, appears to be an effective and safe option for pediatric Crohn’s disease (CD), but the potential risk of opportunistic infection and autoimmune disease is of particular concern. We present the first pediatric case of viral encephalitis (VE) in a 16-year-old boy with CD following treatment with ADA. A 16-year-old CD patient developed symptoms such as fever, dizziness, drowsiness, and blurred vision after three months of ADA treatment. Fundoscopic examination demonstrated pronounced edema of the optic disc in both eyes. The lumbar puncture revealed a marked elevation in intracranial pressure and pathogen analysis of the cerebrospinal fluid identified an HHV-7 infection using next generation sequencing. Following discontinuation of ADA and treatment with acyclovir, the child’s symptoms largely subsided. Literature search identified 11 published CD adult patients with neurological complications related to ADA treatment, while 7 cases were diagnosed with autoimmune encephalitis and 4 with Guillain-Barré syndrome. This report presents the first pediatric CD patient with treatment of ADA, during which VE manifested. Although neurological adverse events associated with ADA are infrequent in pediatrics, vigilant monitoring for neurological symptoms, particularly for infections, remains critical.

## 1 Introduction

Inflammatory bowel disease (IBD) is a chronic systemic inflammatory disorder comprising three primary disease entities: Crohn’s disease (CD), ulcerative colitis (UC), and indeterminate colitis (1). The advent of biological therapy has significantly altered the management of IBD, with anti-tumor necrosis factor (TNF) agents emerging as a cornerstone of treatment (2). However, the use of TNF-α inhibitors may be associated with the reactivation of latent viruses or the onset of new infections (3). Adalimumab (ADA), a fully humanized monoclonal antibody, is extensively utilized in the management of CD and is purported to exhibit reduced immunogenicity and an improved safety profile compared to other agents (4). Neurological manifestations directly attributable to treatment are infrequent, making it challenging to establish a definitive causal link. The incidence of viral encephalitis (VE) as a complication of such therapies is exceedingly rare, with no documented cases in the medical literature involving CD patients treated with ADA. Herein, we present the case of a 16-year-old male with CD, in whom ADA therapy was complicated by the development of VE.

## 2 Case description

A 16-year-old boy with CD, treated with ADA (40 mg subcutaneously every other week) for three months, presented with acute fever, dizziness, drowsiness, blurred vision, frontal and nuchal pain, and diplopia for one day. He had missed two scheduled doses due to a preceding upper respiratory tract infection. The patient was admitted for urgent evaluation of suspected central nervous system infection or inflammation.

**Figure 1:**
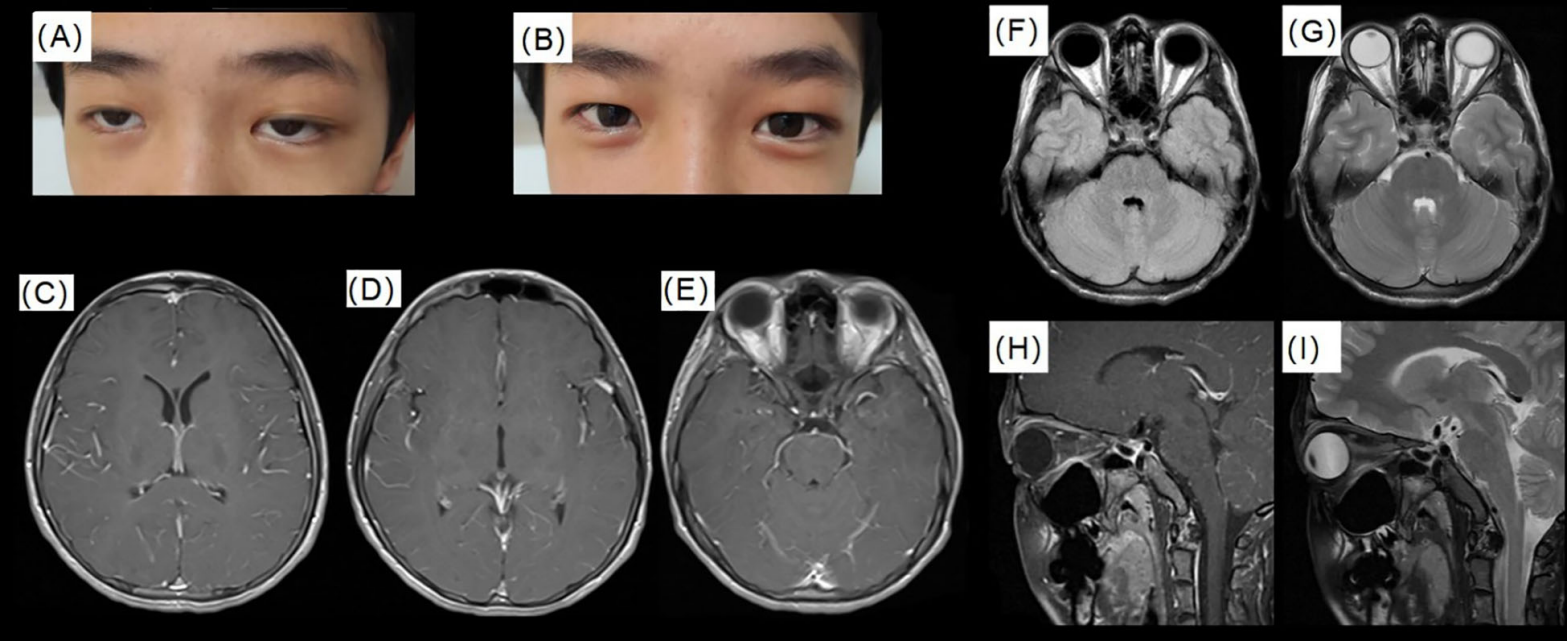
The patient’s eyes, intracranial pressure and cranial MRI. **(A)** Illustrates the condition of the child’s eyes upon admission; **(B)** Depicts the condition of the child’s eyes following two weeks of treatment with Acyclovir; **(C–E)** T1 post contrast images around temporal lobes; **(F–I)** display cranial and orbital MRI findings, which reveal a small amount of fluid accumulation in the optic nerve sheath bilaterally (near the scleral attachment, with a width of less than 1 mm), partial empty sella turcica.

The patient measured 170 cm in height (−1~0SD) and weighed 42 kg (−3~−2SD), resulting in a body mass index (BMI) of 14.53 kg/m² (−3~−2SD). Upon examination, the patient presented with mild somnolence and poor mental status. The neck was supple, with slight pain noted upon movement. Ocular motility was intact, although diplopia was present at a certain visual level (**Figure 1A**). Physical examinations of the cardiovascular, respiratory, and abdominal systems revealed no abnormalities. The muscle strength and tone in the limbs were within normal limits, and there were no indications of meningeal irritation; the remainder of the neurological examination was unremarkable.

**Figure 2:**
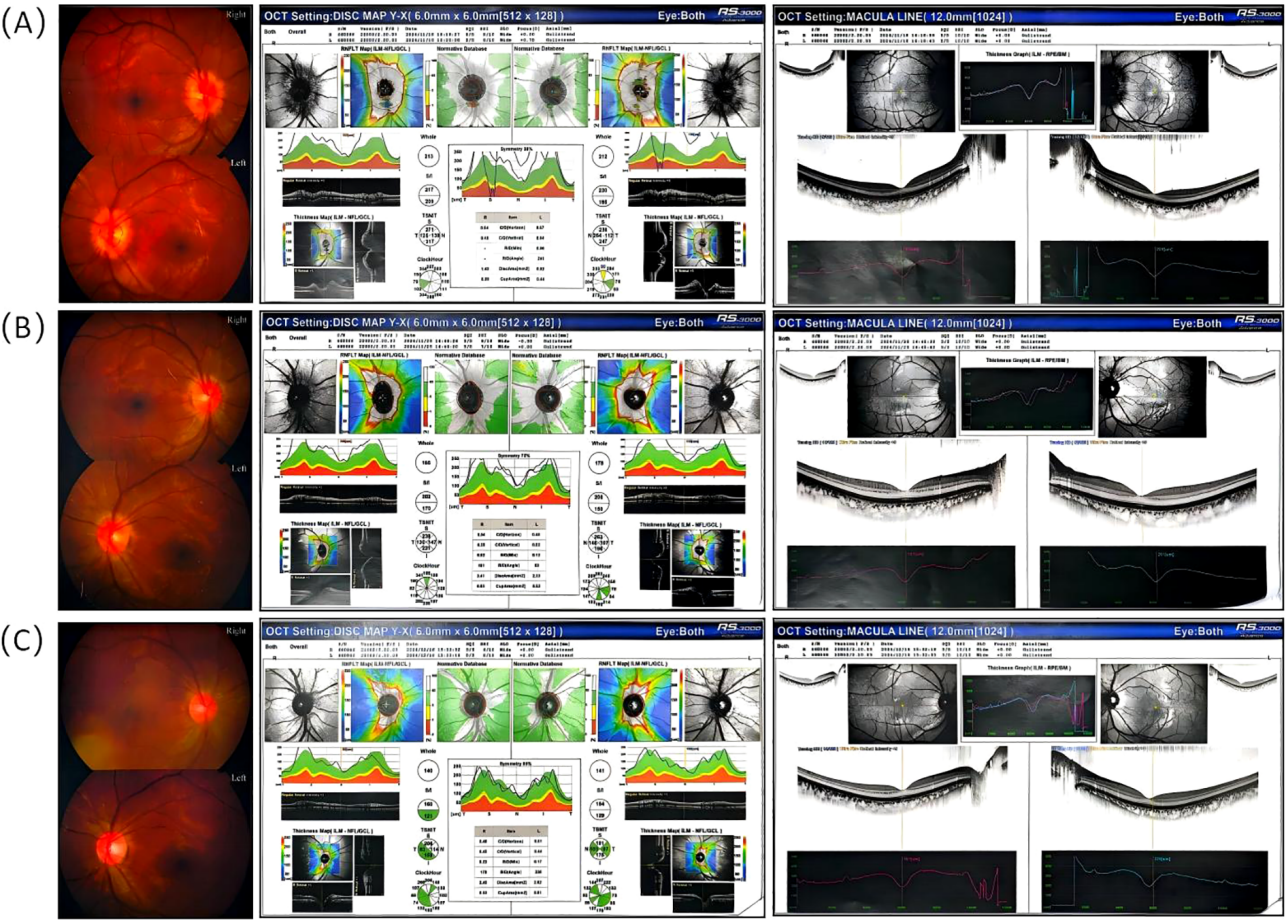
Fundus examination of the child before and after treatment. **(A)** Initial fundus examination prior to treatment revealed significant optic disc edema in both eyes. **(B)** One week post-treatment, without the administration of Acyclovir, there was an observable improvement in the optic disc edema. **(C)** Three weeks following treatment, with Acyclovir administered for two weeks, the optic nerve boundary became distinct and the edema fully resolved.

Initial blood tests revealed no significant abnormalities (**Table 1**). Tests for Influenza A and B were negative, while IgG antibodies for Epstein-Barr virus, herpes simplex virus type I, rubella virus, and Toxoplasma were positive. No other abnormalities were detected in the additional tests. Magnetic Resonance Imaging (MRI) of the cerebral and orbital regions revealed a minor accumulation of fluid within the optic nerve sheath bilaterally, located near the scleral attachment, with the fluid accumulation measuring less than 1 mm in width. Additionally, a partially empty sella turcica was observed, while no significant abnormalities were detected in the remaining structures (**Figure 1**). Fundoscopic examination demonstrated pronounced edema of the optic disc in both eyes (**Figure 2A**). The initial lumbar puncture revealed a marked elevation in intracranial pressure (295 mmH2O), and an increased white blood cell count in the CSF (**Table 1**). However, no pathogen evidence was found in the cerebrospinal fluid microbiology culture at the hospital. To identify the pathogen, the second lumbar puncture was performed and indicated a further increase in intracranial pressure (330 mmH2O), while next-generation sequencing (NGS) was used and detected human herpesvirus type 7 (HHV-7, sequence number: 7766; relative abundance: 99.94%; confidence level of the detection: 99%; genomic coverage: 37.69%).

**Table 1:** The correlation of cerebrospinal fluid and blood routine indexes before and after treatment.

Index	Pre-therapy	1 week (ADA was discontinued and acyclovir was not used)	1042 weeks post-treatment (Acyclovir was used)	1043 weeks post-treatment (Acyclovir was used)
Blood				
WBC×10^9/L	5.82	6.2	4.68	5.43
RBC×10^12/L	5.14	4.8	4.87	4.7
PLT ×10^9/L	345	294	260	273
HGB (g/L)	153	143	147	142
N%	56.1	57.1	63.3	57.7
L%	32.8	31.9	25.2	31
hs-CRP (mg/L)	0.65	<0.2	0.22	0.40
ESR (mm/h)	19.39	–	–	–
K^+^ (mmol/L)	4.02	3.8	3.83	4.38
Na^+^ (mmol/L)	131.92	135.92	134.11	139.74
Cl^-^ (mmol/L)	95.88	98.82	94.48	102.62
Ca^2+^ (mmol/L)	2.37	2.45	2.35	2.59
CSF				
Intracranial pressure (mmH_2_O)	295	330	190	90
Cl of CSF (mmol/L)	124.38	126.10	125.50	124.25
Glu of CSF (mmol/L)	3.69	3.58	3.04	3.29
Total protein of CSF (g/L)	0.73	0.44	0.35	0.33
WBC of CSF (×10^6/L)	158	63	35	22
Mononuclearcell of CSF	0.962	1.000	0.989	0.983
Multiple nuclear cells of CSF	0.038	0.000	0.011	0.017
RBC of CSF (×10^12/L)	0.000	0.000	0.000	0.000
Pandy test of CSF	–	–	–	–
Color of CSF	colorless	colorless	colorless	colorless
Pellucidity of CSF	transparent	transparent	transparent	transparent
Etiology of CSF (Germiculture)	Normal	Normal	Normal	Normal
Etiology of CSF (NGS)	NA	HHV7 (Sequence number 7766)	EBV(Sequence number 35)	EBV (Sequence number10)

CSF, Cerebrospinal fluid; WBC, White blood cell; RBC, Red blood cell; PLT, Platelet; HGB, Hemoglobin; N, Neutrophile granulocyte; L, Leukomonocyte; hs-CRP, Hyper-sensitive C-reactive protein; ESR, Erythrocyte sedimentation rate; NGS, Next generation sequencing; NA, Not Available.

Normal reference range of CSF: Intracranial pressure <100mmH_2_O; Cl 120~130mmol/L; Glu CSF 2.5~4.5mmol/L; Total protein 0.15~0.45g/L; WBC<15×10^6/L; RBC = 0.

Normal reference range of peripheral blood: WBC 4~10×10^9/L; RBC 4~5.5×10^12/L; PLT 100~300× 10^9/L; HGB 120~160g/L; N% 50~70%; L% 20~40%; hs-CRP 0~10mg/L; ESR 0~15mm/h; K^+^ 3.5~5.3 mmol/L; Na^+^ 136~145 mmol/L; Cl^-^ 96~108mmol/L; Ca^2+^ 2.08~2.80 mmol/L.

Based on these findings and additional diagnostic evaluations, it was concluded that the HHV-7-related VE was induced by the use of ADA. ADA was discontinued. The patient was subsequently transitioned to exclusive enteral nutrition and administered oral salicylate to reduce bowel inflammation. Meanwhile, the patient was initiated on acyclovir therapy (10mg/kg, q8h, 2 weeks intravenous and 1 week orally). Following the aforementioned therapeutic interventions, the patient’s intracranial pressure returned to normal levels (**Table 1**), the optic nerve edema completely resolved (**Figures 2B, C**), and the patient’s blurred vision showed improvement (**Figure 1B**). Additionally, HHV-7 was not detected in the CSF after acyclovir therapy. At present, the patient is treated with oral thalidomide (75mg, daily) to reduce intestinal inflammation. We followed up once a month for half a year and the intestinal inflammation of the patient has been reduced compared with before. The patient is asymptomatic and the latest blood hs-CRP and ESR are within the normal range.

## 3 Discussion

In this study, we present a case of a 16-year-old CD patient who developed symptoms such as fever, dizziness, drowsiness, and blurred vision after three months of ADA treatment. The association between ADA and VE is supported by the following evidence including drowsiness, resolution of symptoms following ADA discontinuation, evidence of CSF infection with HHV-7, and the effectiveness of acyclovir treatment. HHV-7 is a recognized neurotropic virus capable of causing encephalitis (5). This case demonstrates that children with CD receiving ADA therapy are at risk of opportunistic central nervous system infections in the context of immunosuppression.

It is well-established that CD is a chronic inflammatory bowel disease characterized by a relapsing-remitting course, involving transmural inflammation that can affect any segment of the gastrointestinal tract (1). ADA is a subcutaneously administered, recombinant, fully human IgG1 monoclonal antibody that binds with high affinity and specificity to human TNF-α, thereby modulating its biological functions and pro-inflammatory effects in CD. ADA is purported to exhibit lower immunogenicity and a more favorable safety profile compared to other agents (4). TNF-α is a proinflammatory cytokine involved in an intricate inflammatory network. TNF-α modulates monocyte differentiation, induces the production of an assortment of chemokines and adhesion molecules, stimulates T-cell proliferation and also promotes T-cell apoptosis and the termination of immune response. At low levels, TNF-α has beneficial effects in the tissue, e.g., an increase in host defense mechanisms against infections; conversely high concentrations may lead to excess inflammation and organ injury (6). The inhibition of ADA may lead to decreased cell-mediated immunity, reduced release of inflammatory mediators, suppressed leukocyte migration, and diminished pathogen clearance capacity, thereby increasing susceptibility to reactivation of latent viruses and opportunistic infections (3). The most common adverse reaction of ADA is respiratory tract infection, while VE in children with CD during ADA administration has not been reported.

Following its approval by the Food and Drug Administration for pediatric CD patients, long-term follow-up data on ADA use in CD patients have become available (4). The most common adverse reaction of ADA is respiratory tract infection. Among the documented neurological disorders associated with anti-TNF-α therapy, central and peripheral demyelinating diseases are the most common adverse reactions, while no reports of VE in pediatric CD patients treated with ADA were found in the WHO adverse reaction database (7). We conducted a review of previously reported cases involving central nervous system symptoms in 15 CD patients undergoing ADA therapy (**Table 2**) (8–22). Notably, all patients were over the age of 20, and 11 developed neurological abnormalities within one year of ADA initiation, predominantly presenting as autoimmune encephalitis (7 cases) and Guillain-Barré syndrome (4 cases). Although TNF-α exhibits pro-inflammatory effects, its deficiency can also lead to impaired myelin-specific T cell responsiveness and prolonged survival of activated T cells (23). Long-term administration of TNF-α antagonists is believed to enhance autoimmune responses by altering antigen-presenting cell function, potentiating T cell receptor signaling, and reducing apoptosis of self-reactive T cells (24). Consequently, TNF-α antagonist therapy may induce or exacerbate harmful tissue inflammatory damage by increasing the number of activated peripheral T cells or disrupting the intrinsic balance of TNF-α and its receptors in the local peripheral nervous system, thereby promoting the development of autoimmune encephalitis and Guillain-Barré syndrome (11, 25, 26). Despite these insights, the pathophysiological mechanisms of other immune-mediated side effects associated with TNF inhibitors remain less clearly understood.

**Table 2:** A review of previously reported cases involving central nervous system symptoms in CD patients undergoing ADA therapy.

Case NO.	Country	Year of publication	Sex	Age	Proto-pathy	The manifestations of Encephalitis	Therapy duration of ADA	MRI/MSA	Complication	Therapeutic Measures	Outcome	Reference
01	Italy	2011	F	53Y	CD	Nuchal headache, dizziness and right superior quadrantopsy	4M	Abnormal	Cerebral vasculitis	Discontinuation Methylprednisolone Prednisone Aspirin	Improvement	Vannucchi V (2011) (8)
02	Spain	2020	F	53Y	CD	Behavioral disorders, hallucinations, seizures, and fever.	6M	Abnormal	Autoimmune Encephalitis	Discontinuation Intravenous steroids Intravenous immunoglobulin	Improvement	Paula Fernández Alvarez(2020) (9)
03	USA	2018	F	58Y	CD	Progressive encephalopathy.	6M	Normal	Anti–NMDAR encephalitis	Discontinuation Methylprednisolone Intravenous immunoglobulin Rituximab	Improvement	Noble GP (2018) (10)
04	Canada	2024	F	46Y	CD	Bizarre behaviors, headaches, reported auditory hallucinations.	6Y	Normal	Anti–NMDAR encephalitis	Discontinuation Methylprednisolone Intravenous immunoglobulin Prednisone Rituximab	Improvement	MacKay S (2024) (11)
05	France	2020	F	20Y	CD	Viral syndrome: pustular lesions, a purpuric skin rash, joint pain.	9M	Abnormal	Henoch-Schönlein purpura with nervous system involvement	Colchicine Intravenous corticosteroids	Improvement	Condamina M (2020) (12)
06	USA	2017	F	52Y	CD	Chronic myelitis - motor and sensory disturbance.	1Y	Abnormal	Autoimmune Encephalitis	Discontinuation Prednisone	Improvement	Barreras P (2017) (13)
07	UK	2014	F	26Y	CD	Intermittent diplopia, clumsy and her left leg weak.	4M	Abnormal	Inflammatory demyelination	Discontinuation Methylprednisolone Plasmapheresis	Slight improvement	Hare NC (2014) (14)
08	UK	2015	F	27Y	CD	Left facial numbness, altered taste sensation, blurred vision, horizontal diplopia on right gaze.	18M	Abnormal	Internuclear ophthalmoplegia	Discontinuation Steroid	Improvement	Drury J(2015) (15)
09	Australia	2017	M	37Y	CD	Acute neurological symptoms with impaired sensation, progressive bilateral lower motor neuron facial weakness.	8M	Abnormal	chronic inflammatory demyelinating polyneuropathy	Discontinuation Intravenous immunoglobulin Steroid	Improvement	Yao A(2017) (16)
10	Brazil	2017	M	64Y	CD	Progressive moderate to severe bilateral symmetrical weakness.	3M	NA	Guillain-Barre syndrome	Discontinuation	Improvement	Cançado GG(2017) (17)
11	Australia	2017	M	37Y	CD	Acute-onset bifacial weakness, mild upper limb weakness and global areflexia.	9M	Normal	Guillain-Barre syndrome	Discontinuation Intravenous immunoglobulin	Slight improvement	Patwala K(2017) (18)
12	Italy	2011	M	71Y	CD	Progressive severe bilateral symmetric weakness of the legs	2M	NA	Guillain-Barre syndrome	Discontinuation Intravenous immunoglobulin Methylprednisolone Plasmapheresis	Slight improvement	Cesarini M(2011) (19)
13	South Korea	2018	F	33Y	CD	weakness of both the upper and lower extremities occurred, sensory loss.	2M	NA	Guillain-Barre syndrome	Continuation of ADA treatment Intravenous immunoglobulin	Improvement	Lee JH(2018) (20)
14	Canada	2013	M	40Y	CD	Constant bifrontal, progressively worsening headaches with photophobia.	6M	NA	Varicella zoster virus meningitis	Discontinuation Intravenous acyclovir	Not effect	Ma C(2013) (21)
15	UK	2021	F	31Y	CD	Headache, fever and an isolated episode of left-sided facial twitching.	10Y	Abnormal	Anti-MOG associated encephalitis with seizures	Empiric antimicrobial therapy: antibiotic and antiviral Levetiracetam Methylprednisolone Prednisolone	Improvement	Stamenova S (2021) (22)

M, Man; F, Feman; Y, Year; M, Mouth; CD, Crohn’s disease; ADA, adalimumab.

A thorough differential diagnosis was imperative given the patient’s presentation with neurological symptoms under immunosuppressive therapy. Infectious etiologies were paramount. Bacterial, tuberculous, or fungal encephalitis were considered less likely due to the absence of systemic sepsis signs, normal serum procalcitonin, colorless CSF, negative initial CSF cultures, and the lymphocytic predominance in CSF. Other viral pathogens, including herpes simplex virus (HSV), varicella-zoster virus (VZV), and Epstein-Barr virus (EBV), were also plausible causes of encephalitis in an immunocompromised host. Given the patient’s recent onset and mild symptoms, the hospital’s testing sensitivity was limited, potentially failing to detect low-concentration pathogens in the CSF. Consequently, a repeat lumbar puncture was performed, and NGS was used to analyze the CSF, which identified HHV-7. Autoimmune-mediated disorders, such as anti-NMDA receptor encephalitis or other autoimmune encephalitis, were also crucial considerations, as they have been reported in association with ADA therapy. The absence of characteristic psychiatric symptoms, movement disorders, seizures, and the lack of specific autoantibodies in the CSF argued against this possibility. Finally, other causes of raised intracranial pressure and encephalopathy, such as metabolic disturbances, toxic exposures, or intracranial space-occupying lesions, were excluded by comprehensive laboratory testing and contrast-enhanced cranial MRI. The clinical symptoms, significant intracranial hypertension, CSF lymphocytic pleocytosis with HHV-7 detection, and response to acyclovir solidified the diagnosis of HHV-7-associated encephalitis as a complication of ADA-induced immunosuppression. After the patient’s symptoms completely resolved, follow-up CSF testing revealed no HHV-7 but detected extremely low levels of EBV. Considering the very low viral load and the patient’s asymptomatic condition, observation was temporarily recommended. After 1 week, the viral load in CSF by NGS further decreased and the subsequent follow-up showed no clinical abnormalities in the patient, which did not support central nervous system EBV infection. EBV could establish life-long persistence and replicate at low subclinical levels in human reservoirs (27, 28). After the onset of encephalitis, patients may experience disruption of the blood-brain barrier, allowing low levels of EBV DNA in the peripheral blood to enter the cerebrospinal fluid. In other studies, low number of sequences EBV reads has also been reported by CSF NGS and similarly considered as a reflection of EBV persistence or cross contamination other than active diseases (29, 30). In addition, persistent CSF pleocytosis were observed after three weeks of treatment and the patient’s clinical symptoms resolved completely. The normalization of CSF parameters may lag behind clinical improvement in viral encephalitis, particularly in immunocompromised hosts (31, 32). Even after effective antiviral therapy and symptom resolution, a mild lymphocytic pleocytosis may persist for several weeks as part of the ongoing intrathecal immune response and antigen clearance (33).

With advancements in science and technology, NGS has become a prevalent tool in clinical pathogen detection (34). In this study, the initial lumbar puncture which was sent for microbial culture of hospital did not yield any pathogen evidence. However, subsequent analysis of cerebrospinal fluid obtained from a second lumbar puncture, using NGS, revealed an infection with HHV-7. Compared to traditional culture, serological detection, and PCR methods, NGS offers superior sensitivity and specificity, enabling the detection of pathogens that are challenging to identify using conventional techniques (34). The HHV-7 pathogen detected by NGS exhibited a relative abundance of 99.94%, indicating it was the predominant nucleic acid signal in the CSF and effectively excluding background contamination. Furthermore, the confidence level of the detection reached 99%, reflecting the analytical reliability of the sequence alignment and taxonomic assignment. Genomic coverage of 37.69% further confirms that the virus was detected across a substantial portion of its genome, rather than through sporadic or non-specific reads. Therefore, the high read count (7766), predominance in sequence profile, broad genome coverage, and high confidence collectively satisfy established criteria for a true positive NGS result in sterile site specimens. While PCR remains a valuable confirmatory tool, the strength of the NGS evidence in this case, coupled with the congruent clinical and laboratory evolution, provided a diagnostically definitive basis for targeted antiviral therapy. While PCR remains a valuable confirmatory tool, the strength of NGS evidence in this case, combined with clinical symptoms and antiviral treatment efficacy, provided the basis for diagnostic certainty. Consequently, NGS facilitates more rapid and accurate pathogen identification, thereby enhancing diagnostic and therapeutic decision-making in clinical practice.

Following resolution of HHV-7 encephalitis, ADA was permanently discontinued due to its probable role in predisposing to opportunistic viral reactivation. For ongoing control of CD, thalidomide was selected as an alternative immunomodulatory agent based on its distinct mechanism of action, which involves modulation of T-cell responses and downregulation of pro-inflammatory cytokines without broad suppression of antiviral immunity. Thalidomide has demonstrated efficacy in pediatric refractory CD and carries a lower reported risk of serious opportunistic infections compared to TNF-α inhibitors (35, 36). Although peripheral neuropathy is a known adverse effect, the low dose (75 mg daily) and close neurological monitoring were deemed acceptable in this context, given the imperative to avoid recurrent CNS infection while maintaining intestinal disease control.

This case carries several important implications for clinicians managing pediatric CD patients with anti-TNF therapy. In pediatric patients receiving ADA therapy, clinicians should remain vigilant for potential central nervous system opportunistic infections and actively investigate pathogens if mild neuropsychiatric warning signs such as low-grade fever, dizziness, or lethargy occur, even in the absence of early severe encephalitis symptoms (e.g., seizures, focal functional deficits, or MRI-confirmed parenchymal lesions). Also, it highlights the indispensable role of advanced microbiological diagnostics (e.g., NGS) in immunocompromised hosts presenting with central nervous system symptoms. When conventional culture fails to reveal abnormalities, NGS can rapidly identify rare or unexpected pathogens, enabling timely and targeted treatment. Finally, this report highlights the need for structured monitoring in pediatric patients on ADA.

In conclusion, we present the first documented instance of a pediatric patient with CD undergoing treatment with ADA, during which VE manifested. For central nervous system alterations of undetermined origin, NGS plays a crucial role in elucidating the etiology and informing therapeutic strategies. Following ADA discontinuation and treatment with acyclovir, the patient’s symptoms such as fever, dizziness, and blurred vision, largely subsided. It is imperative to closely monitor neurological symptoms and opportunistic infections in CD patients receiving ADA therapy to optimize therapeutic efficacy and minimize the risk of complications.

## Data Availability

The original contributions presented in the study are included in the article/supplementary material. Further inquiries can be directed to the corresponding authors.
